# Bioactive Chitosan Films from Shrimp and Crab Shells: Physicochemical and Functional Properties Associated with Biological Source and Processing History

**DOI:** 10.3390/polym18212761

**Published:** 2026-11-06

**Authors:** Mónica Alejandra Carrazco-Rivera, Laura Leticia Barrera-Necha, José Luis Trejo-Espino, Pedro Lina-Manjarrez, Jaime Lizardi-Mendoza, Karla Guadalupe Martínez-Robinson, Victor Manuel Orozco-Carmona, José Luis Acosta-Rodríguez

**Affiliations:** 1 Centro Interdisciplinario de Investigación para el Desarrollo Integral Regional Unidad Sinaloa, Instituto Politécnico Nacional, Guasave 81100, Mexico; mcarrazcor2000@alumno.ipn.mx; 2 Centro de Desarrollo de Productos Bióticos, Instituto Politécnico Nacional, Yautepec de Zaragoza 62731, Mexico; lbarrera@ipn.mx (L.L.B.-N.); jtrejo@ipn.mx (J.L.T.-E.); 3 Centro Interdisciplinario de Investigaciones y Estudios Sobre Medio Ambiente y Desarrollo, Instituto Politécnico Nacional, Ciudad de México 07340, Mexico; plina@ipn.mx; 4 Centro de Investigación en Alimentación y Desarrollo, Hermosillo 83304, Mexico; jalim@ciad.mx (J.L.-M.); karlagm@ciad.mx (K.G.M.-R.); 5 Centro de Investigación en Materiales Avanzados, Chihuahua 31136, Mexico; victor.orozco@cimav.edu.mx

**Keywords:** chitosan films, crustacean waste, biological origin, chemical–microbiological extraction, molecular weight, antibacterial activity, water absorption, bioactive films

## Abstract

Chitosan films are promising materials for food-related applications, but their performance depends on polymer characteristics. This study compared films from hybrid chemical–microbiological shrimp (CM-Shr) and crab (CM-Crb) chitosans, chemically extracted crab chitosan (Ch-Crb), and high- (Ref-H) and low-molecular-weight (Ref-L) reference chitosans. Films were prepared using 1.5% (*w*/*v*) chitosan in 1% (*v*/*v*) acetic acid with glycerol at 30% (*w*/*w*, relative to dry chitosan). Chitosans were characterized by degree of deacetylation, molecular weight, moisture, ash, nitrogen and ATR-FTIR; films were assessed for thermal, morphological, hydration, mechanical, optical, and antibacterial properties. ATR-FTIR indicated differences in amino, residual N-acetyl, and hydrogen-bonded environments. At 15 min, CM-Crb and Ch-Crb exhibited lower apparent water absorption (34.5 ± 1.2% and 32.6 ± 0.7%) than Ref-H (51.8 ± 1.9%). Antibacterial inhibition beyond the 6 mm film-disk diameter was absent at 1.5% and predominantly detected for CM-Crb and Ch-Crb at 3.0–4.5% (*w*/*v*) chitosan in the film-forming solutions. CM-Crb exhibited tensile strength of 52.1 ± 4.3 MPa, the highest elongation at break (3.7 ± 0.3%) and UV blocking at 340 nm (35%). Film performance was associated with the physicochemical profiles and processing history of the starting chitosans.

## 1. Introduction

The increasing environmental impact of petroleum-based plastic packaging has intensified interest in biodegradable polymers obtained from renewable resources [1,2]. Chitosan, an aminopolysaccharide produced by partial deacetylation of chitin, is of particular interest because of its biodegradability, film-forming capacity, and antimicrobial properties [3,4]. Chitin occurs in crustacean shells, insect exoskeletons, and fungal cell walls [5], and its recovery from food-processing residues provides a route for converting underutilized biomass into polymeric materials [6].

Global aquaculture production of crustaceans reached 12.8 million tonnes in 2022 [7], generating substantial quantities of shells and cephalothorax residues. These materials contain chitin together with proteins, minerals, pigments, and other components and therefore represent an important feedstock for chitin and chitosan recovery [8,9].

Various technologies have been developed for chitin and chitosan recovery, with chemical and biological approaches being among the most extensively investigated. Conventional chemical extraction typically involves acid demineralization followed by alkaline deproteinization and deacetylation and remains widely employed because of its high efficiency and relatively short processing time. Nevertheless, harsh acidic and alkaline conditions may promote polymer-chain degradation and reduce molecular weight, while hindering the recovery and valorization of protein and mineral fractions and generating chemically loaded effluents [10,11]. In contrast, biological extraction, based on microbial fermentation and/or enzymatic treatments, has been explored as an alternative for specific processing stages, particularly deproteinization, with the potential to reduce the severity of chemical treatments during these stages. However, this potential reduction is partial in hybrid approaches that retain conventional alkaline deacetylation, and longer processing times and process-control requirements remain important limitations [11,12,13].

Chitosan-based films and coatings have been widely investigated for food packaging and agricultural applications due to their film-forming ability, mechanical and barrier properties, biodegradability, and inherent antimicrobial potential [14,15]. The physicochemical and functional performance of chitosan films is strongly influenced by intrinsic polymer characteristics, particularly molecular weight and degree of deacetylation, as well as by film-forming conditions, including the nature of the solvent acid, polymer and acid concentrations, the incorporation of additives, and drying conditions, while the biological source and extraction conditions can further determine the molecular and structural characteristics of the resulting chitosan [16,17,18].

Although chitosan films from different biological sources and extraction processes have been widely investigated, comparison across studies is complicated because studies differ in biological source, extraction conditions, molecular characteristics, plasticizer content, film thickness, and drying conditions [16,17,18,19]. Consequently, differences in film performance cannot readily be assigned to a single material characteristic. While the effects of molecular weight and degree of deacetylation on chitosan properties have been investigated [20,21], the relationship between the physicochemical profiles of chitosans with different biological sources and extraction approaches and their film performance under standardized formulation conditions warrants further investigation.

Accordingly, this study aimed to compare the physicochemical and functional properties of films prepared from chitosans differing in biological source, processing history, and molecular characteristics within a standardized film-forming framework. Specifically, chemical–microbiologically extracted shrimp and crab chitosans were compared with chemically extracted crab chitosan and commercial references. The resulting films were evaluated in terms of surface morphology, thermal behavior, apparent water absorption, mechanical performance, optical properties, and antibacterial activity to examine how differences in the physicochemical profiles of the starting chitosans are associated with film performance, without attributing these differences to any single material or processing variable.

## 2. Materials and Methods

### 2.1. Materials

Chitosans from two biological origins (shrimp, *Litopenaeus vannamei*, and crab, *Callinectes bellicosus*) and obtained through different extraction approaches were evaluated in this study. The experimentally produced chitosans comprised chemical–microbiological chitosan from shrimp (CM-Shr), chemical–microbiological chitosan from crab (CM-Crb), and chemically extracted chitosan from crab (Ch-Crb). Crab was selected as the primary biological model to allow comparison of chitosans obtained from the same crustacean source through two different extraction approaches (CM-Crb and Ch-Crb), whereas shrimp-derived CM-Shr was included to evaluate the hybrid chemical–microbiological approach using a different crustacean source. This material selection was intended as a comparative framework and not as a fully factorial design separating the independent effects of biological source and extraction approach.

Two commercial shrimp chitosans supplied by Sigma-Aldrich (St. Louis, MO, USA), differing in molecular weight, were used as reference materials: high-molecular-weight commercial chitosan and low-molecular-weight commercial chitosan. Ref-H (310–375 kDa), with a manufacturer-reported degree of deacetylation (DDA) ≥75%, was deliberately selected as a high-molecular-weight benchmark to broaden the range of polymer characteristics evaluated and provide a reference substantially different from the experimental chitosans (~100–110 kDa). Ref-H was used as the primary commercial reference for the structural, thermal, and optical characterization of the films. Ref-L was additionally included in the dynamic functional assays (apparent water absorption, antibacterial activity, and mechanical performance) as a lower molecular weight commercial reference (50–190 kDa), with a manufacturer-reported DDA of 75–85%, enabling comparisons of functional performance across chitosans with partially overlapping reported molecular-weight values, without isolating the independent contribution of molecular weight.

Glacial acetic acid (analytical grade), glycerol (≥99%), phosphate buffer (pH 7.4), hydrochloric acid (HCl), sodium hydroxide (NaOH), methyl orange, and sodium acetate were purchased from Sigma-Aldrich (St. Louis, MO, USA). All reagents were used without further purification.

### 2.2. Chitosan Production and Extraction Approaches

Chitosans were obtained from shrimp and crab processing residues using either a hybrid chemical–microbiological extraction approach or a conventional chemical extraction approach. Prior to extraction, the crustacean residues were washed, dried, milled, and sieved to obtain a homogeneous powdered raw material.

For the hybrid chemical–microbiological extraction approach, used to obtain CM-Shr from shrimp and CM-Crb from crab, demineralization was performed chemically, while the core principle of the hybrid approach relied on microbiologically mediated deproteinization, thereby reducing the reliance on harsh thermal-alkaline treatments during this processing stage. The hybrid process therefore produces a chitin-rich intermediate, which is subsequently converted to chitosan by conventional chemical deacetylation under alkaline conditions. Thus, the microbiological stage is used for deproteinization and does not replace the chemical deacetylation step. The process is currently the subject of a pending patent application; therefore, specific proprietary operational parameters cannot be disclosed at this stage. Nevertheless, the principal processing sequence and the role of each stage are provided here to clarify the experimental approach used to obtain the chitosans evaluated in this study. The conditions used correspond to the laboratory-scale process developed to produce these experimental chitosans and should not be interpreted as a fully optimized industrial production process.

For comparison, Ch-Crb was obtained from crab residues using a conventional chemical extraction approach based on the classical acid–alkali methodology for chitin isolation [22], followed by alkaline deacetylation to obtain chitosan. Demineralization was performed with 3% (*w*/*v*) HCl at a solid-to-liquid ratio of 1:10 (*w*/*v*), under constant agitation (100 rpm) at 25 °C for 1 h. Chemical deproteinization was subsequently performed with 50% (*w*/*w*) NaOH at a solid-to-liquid ratio of 1:10 (*w*/*v*), under constant agitation (100 rpm) at 110 °C for 3 h, following the alkaline treatment conditions reported by Said Al Hoqani et al. [23] for chitin extraction from crustaceans, with the aim of facilitating protein removal. However, the Fourier-transform near-infrared (FT-NIR) analysis reported in that study covered the 14,000–4000 cm^−1^ range, which excludes the characteristic mid-infrared bands commonly used to assess chitin deacetylation. Consequently, partial deacetylation during this severe alkaline treatment cannot be excluded, although its occurrence and extent were not independently evaluated at this processing stage. The recovered chitin was then deacetylated with 50% (*w*/*w*) NaOH at a solid-to-liquid ratio of 1:10 (*w*/*v*), under constant agitation (100 rpm) at 100 °C for 3 h. After each stage, the solid fraction was washed with distilled water until neutral pH, filtered, and dried at 60 °C until constant weight. The complete chemical extraction procedure was independently performed on three separate samples under the same experimental conditions.

### 2.3. Characterization of Chitosan

#### 2.3.1. Degree of Deacetylation

The degree of deacetylation of the experimentally produced chitosans was determined by acid–base titration following the method described by Dutta and Priyanka [24], with minor modifications. Briefly, 1 g of chitosan was dissolved in 100 mL of 0.1 M HCl under continuous stirring for 2 h until complete dissolution. A 30 mL aliquot of the resulting chitosan solution was transferred to a conical flask, and 2–3 drops of methyl orange indicator were added. The solution was titrated with 0.1 M NaOH until the endpoint was reached, as indicated by a color change from pink to yellow-orange (within the methyl orange transition range of pH 3.1–4.4). The volume of NaOH consumed was recorded and used to calculate the DDA. The DDA was calculated according to Equation (1):

(1) $$\mathrm{DDA}\left(\%\right)=\frac{\left(V_{1}-V_{2}\right)\times 16}{V_{1}\times 9.94\times m}\times 100$$

where *V*_1_ is the volume of chitosan solution in 0.1 M HCl used for titration (mL), *V*_2_ is the volume of 0.1 M NaOH consumed during titration (mL), 16 is the equivalent mass of the amino group (–NH_2_), 9.94 represents the theoretical amino-group content of fully deacetylated chitosan (%), and *m* is the mass of chitosan contained in the aliquot (g). The determination was performed in triplicate for each chitosan sample.

#### 2.3.2. Viscosity-Average Molecular Weight

The viscosity-average molecular weight (MW) of the experimentally produced chitosans was determined by dilute-solution capillary viscometry using an Ubbelohde viscometer. Chitosan solutions were prepared in 0.3 M acetic acid/0.2 M sodium acetate, and measurements were performed at 25 °C. The flow times of the solvent and chitosan solutions at different concentrations were measured in triplicate, with a maximum variation of ±1 s among readings. Intrinsic viscosity [*η*] was determined from the concentration dependence of reduced and inherent viscosities using the Huggins and Kraemer relationships. The MW was subsequently calculated using the Mark–Houwink Equation (2):

(2) $$[\eta ]=KM_{v}^{a}$$

where [*η*] is the intrinsic viscosity, *M_v_* is the viscosity-average molecular weight, and *K* = 0.076 and *a* = 0.76 are the Mark–Houwink parameters corresponding to the chitosan–solvent system and experimental conditions [25].

#### 2.3.3. Moisture and Ash Content

Moisture content was determined gravimetrically in triplicate by oven-drying chitosan samples at 105 °C to constant weight. The ash content of the experimentally obtained chitosans was determined by the incineration method described by Rosmawati et al. [26]. Briefly, chitosan samples were placed in pre-weighed porcelain crucibles and incinerated in an electric muffle furnace at 500 °C for 45 min, followed by heating at 900 °C for 1 h. After incineration, the crucibles were cooled and weighed. Ash content was calculated according to Equation (3):

(3) Ash content %=m2−m1 m×100

where *m* is the initial mass of the chitosan sample (g), *m*_1_ is the mass of the empty porcelain crucible (g), and *m*_2_ is the mass of the crucible plus the remaining ash after incineration (g).

#### 2.3.4. Total Nitrogen Content

Total nitrogen content of chitosans was determined in triplicate using the Kjeldahl method [26]. Samples (approximately 100 mg) were digested with 5 mL of concentrated H_2_SO_4_ and a catalyst tablet at 350 °C for 70 min, followed by steam distillation using a Kjeltec 8200 system (FOSS, Hillerød, Denmark) and titration with 0.1 N HCl. Total nitrogen content (%) was calculated using Equation (4) and expressed on a dry-weight basis:

(4) Nitrogen content %=V1−V2× N×14.007 m×100

where *V*_1_ and *V*_2_ are the volumes of HCl consumed during titration of the sample and blank (mL), respectively; *N* is the normality of the HCl solution; 14.007 is the molar mass of nitrogen (g/mol); and *m* is the dry sample mass (mg).

#### 2.3.5. Fourier Transform Infrared Spectroscopy (FTIR)

The starting chitosans were characterized by Fourier transform infrared spectroscopy (FTIR) using a Nicolet iS50 FTIR spectrometer (Thermo Fisher Scientific, Waltham, MA, USA) equipped with an attenuated total reflectance (ATR) accessory. Spectra were recorded over the 4000–500 cm^−1^ range using 32 scans at a spectral resolution of 2 cm^−1^. Spectra were collected directly from the powdered chitosan samples using the ATR accessory.

### 2.4. Preparation of Chitosan Films by Solvent Casting

Chitosan films were prepared using the solvent-casting technique as described previously [27], with slight modifications. Each chitosan type was dissolved at 1.5% (*w*/*v*) in 1% (*v*/*v*) acetic acid under continuous magnetic stirring for 2 h. Glycerol at 30% (*w*/*w*, based on chitosan dry weight) was added as a plasticizer, and the mixture was stirred for an additional 30 min. This glycerol proportion was selected to provide adequate plasticization and was maintained constant across all formulations. A volume of 28.4 mL of each film-forming solution was cast into leveled circular polystyrene Petri dishes (area = 56.8 cm^2^). Finally, the films were dried at 40 °C for 48 h in an oven, then carefully removed from the casting surfaces and stored in a desiccator until further characterization. Based on the nominal formulation, chitosan accounted for approximately 76.9% (*w*/*w*) of the combined chitosan and glycerol mass, regardless of the chitosan concentration in the film-forming solution. This calculation excludes any residual acetic acid, or other species potentially retained after drying.

### 2.5. Thermogravimetric Analysis (TGA)

Thermal stability was evaluated using a TGA Q600 analyzer (TA Instruments, New Castle, DE, USA). Approximately 8–10 mg of each film sample was heated from 30 to 800 °C at a heating rate of 10 °C min^−1^ under an air atmosphere at a flow rate of 50 mL min^−1^. Derivative thermogravimetric (DTG) curves were obtained from the TG curves, and the degradation temperature (Td) of each thermal event was identified from the corresponding DTG maximum.

### 2.6. Scanning Electron Microscopy (SEM)

The top surface morphology of the chitosan films was examined by scanning electron microscopy (SEM) using a Hitachi TM3000 scanning electron microscope (Hitachi High-Tech Corporation, Tokyo, Japan). Prior to analysis, the films were dried in an oven at 35 °C for 48 h and coated with a thin layer of gold using an ion sputter coater (E-1010, Hitachi, Japan). The films were examined using secondary electron imaging (SE/LEI) at an accelerating voltage of 2.0 kV and a working distance of approximately 8 mm. Surface micrographs were acquired at magnifications of ×2500 and ×5000 to evaluate film homogeneity, porosity, and surface morphological features. Approximate dimensions of visible pores and surface features were estimated directly from the scale bars of the SEM micrographs.

### 2.7. Apparent Water Absorption Behavior

Apparent water absorption behavior was measured using a method adapted from Gong et al. [28]. Four film samples (2 cm × 2 cm) from each treatment (*n* = 4) were dried at 60 °C to constant weight and weighed to obtain the initial dry weight (P_1_). The samples were then immersed in phosphate buffer (pH 7.4) at 37 °C. Apparent water absorption was determined after 15, 30, 60, 120, 180, 240, 480, 720, and 1440 min of immersion. At each measurement time, the same samples were removed from the buffer, gently blotted to remove excess surface water, weighed (P_2_), and subsequently returned to the buffer until the next measurement. Apparent water absorption (WA) was calculated according to Equation (5):

(5) $$\mathrm{Apparent water absorption}\left(\%\right)=\frac{P_{2}-P_{1}}{P_{1}}\times 100$$

where P_1_ represents the experimentally measured initial dry mass of each individual film specimen before immersion, and P_2_ represents the wet mass of the same specimen at each sampling time after removing excess surface liquid. The same film specimens were repeatedly weighed throughout the immersion period. The calculated values represent apparent water absorption, as potential losses of soluble film components were not independently quantified.

### 2.8. Antibacterial Properties

Antibacterial activity was evaluated using an agar disk-diffusion assay [29], with modifications. For the antibacterial assay, additional films were prepared from film-forming solutions containing 3.0% and 4.5% (*w*/*v*) chitosan in 1% (*v*/*v*) acetic acid, following the same procedure described in Section 2.4. Thus, films prepared from film-forming solutions containing 1.5%, 3.0%, and 4.5% (*w*/*v*) chitosan were evaluated for each chitosan type. Ref-L was additionally included as a commercial low-molecular-weight reference in the antibacterial assays to evaluate whether the antibacterial response observed for the experimentally obtained chitosans could be associated with MW.

Bacterial suspensions of *Staphylococcus aureus* ATCC 29213, *Salmonella Typhimurium* ATCC 14028 and *Escherichia coli* ATCC 25922 were adjusted to a 0.5 McFarland standard (approximately 1 × 10^8^ CFU/mL). Müeller–Hinton agar (BD Bioxon, Cuautitlán Izcalli, Mexico) plates were inoculated using sterile cotton swabs to obtain a uniform bacterial lawn over the entire agar surface. Circular samples of the chitosan films (6 mm in diameter) were placed on the inoculated agar plates and incubated at 37 °C. Inhibition zones were measured after 24 h of incubation and expressed as the total inhibition-zone diameter (mm), including the 6-mm film disk. Three independent replicates were evaluated for each combination of chitosan type, film-forming solution chitosan concentration, and bacterial strain (*n* = 3).

Ciprofloxacin (CIP) was used as a positive growth-inhibition control at strain-specific concentrations selected from preliminary inhibition assays to produce detectable growth inhibition (0.5 µg/mL for *S. Typhimurium*, 0.375 µg/mL for *E. coli*, and 7.5 µg/mL for *S. aureus*). A volume of 20 µL of the corresponding CIP solution was applied to each disk. The disk-diffusion assay was used as a comparative method to evaluate inhibition extending beyond the film disk and therefore reflects antibacterial effects associated with species able to diffuse from the film into the agar. Accordingly, the absence of an inhibition zone beyond the 6 mm film-disk diameter was interpreted as the absence of detectable inhibition under the conditions of this assay.

### 2.9. Mechanical Tests

Mechanical properties, including tensile strength (TS), elongation at break (EAB) and Young’s modulus (YM), of the chitosan films were evaluated using a universal testing machine (Instron 34TM-50, Instron, Norwood, MA, USA). Three specimens from each film formulation were cut into 1-inch-wide strips and tested at a constant crosshead speed of 5 mm/min until rupture, using an initial grip separation of 50 mm. Tests were performed at 23 °C and 40% relative humidity. Force and displacement were continuously recorded during testing. TS (MPa) was calculated as the maximum force divided by the initial cross-sectional area of the specimen. EAB (%) was calculated as the displacement at failure divided by the initial grip separation and expressed as a percentage. YM (GPa) was determined from the slope of the initial linear region of the stress–strain curve. Film thickness was measured using a flat-foot thickness gauge (Mitutoyo, Japan) with a resolution of 0.1 µm.

### 2.10. Optical Tests

The optical properties of the chitosan films were evaluated using a UV–Vis spectrophotometer (UV-2600, Shimadzu, Kyoto, Japan) equipped with UVProbe software (version 2.42). Spectral transmittance was recorded over the wavelength range of 200–800 nm. Total light transmittance and actual diffused light were determined over the visible region (400–700 nm), while UV-blocking capacity was determined at 340 nm. Results were expressed as total light transmittance (%), UV blocking at 340 nm (%), and actual diffused light (%). Film thickness was measured using a flat-foot thickness gauge (Mitutoyo, Kawasaki, Kanagawa, Japan) with a resolution of 0.1 µm. A single measurement was obtained for each film formulation; therefore, the optical results were analyzed descriptively.

### 2.11. Statistical Analysis

Statistical analyses were performed using Statgraphics Centurion 19 (Statgraphics Technologies, Inc., The Plains, VA, USA). Data are expressed as mean ± standard deviation. For apparent water absorption, values obtained at 15 min were analyzed using one-way analysis of variance (ANOVA), with film type as the fixed factor, followed by Tukey’s honestly significant difference (HSD) test for multiple comparisons (*p* < 0.05). The 15-min measurement, corresponding to the initial maximum water uptake observed for all films, was selected for inferential comparison among film types, whereas subsequent measurements were used to describe the temporal hydration profiles.

Antibacterial data were initially summarized as mean ± standard deviation for each chitosan formulation, film-forming solution chitosan concentration, and bacterial strain. Formulations exhibiting no inhibition beyond the 6 mm film-disk diameter (6.00 ± 0.00 mm) were classified as showing no detectable inhibition (ND) and reported descriptively. For the subset comprising CM-Crb, Ch-Crb, and the positive control CIP at film-forming solution chitosan concentrations of 3.0% and 4.5% (*w*/*v*), separate two-way ANOVAs were performed for each bacterial strain (*S. aureus*, *S. Typhimurium*, and *E. coli*), with treatment, concentration, and their interaction as fixed factors and total inhibition-zone diameter (mm) as the dependent variable. Normality of residuals and homogeneity of variances were assessed using the Shapiro–Wilk and Levene tests, respectively. Pairwise comparisons among treatments exhibiting detectable inhibition within each bacterial strain and concentration were performed using Tukey’s HSD test. Statistical significance was established at *p* < 0.05.

Mechanical properties, including tensile strength, elongation at break, and Young’s modulus, were analyzed separately using one-way ANOVA, with chitosan type as the fixed factor. When the overall ANOVA indicated significant differences, Tukey’s HSD test was used for pairwise comparisons among film formulations (*p* < 0.05). Homogeneity of variances was assessed using Levene’s test.

## 3. Results and Discussion

### 3.1. Characterization of the Starting Chitosans

#### 3.1.1. Physicochemical Characterization of Chitosans

The biological origin, extraction approach, DDA, MW, ash content, total nitrogen, and moisture content of the experimentally produced chitosans are summarized in Table 1. The chitosans exhibited differences in DDA but relatively similar MW values (~100–110 kDa), while ash contents remained below 1% in all experimentally produced samples. The low ash contents indicate limited residual inorganic material and are consistent with effective mineral removal during processing. Total nitrogen contents ranged from 6.5 to 6.9% (dry basis), while moisture contents ranged from 6.6 to 6.9%. Since nitrogen is an intrinsic component of the chitosan structure, total nitrogen content should not be interpreted as a direct measure of residual protein. The similar MW values obtained through both chemical and chemical–microbiological extraction suggest that the final molecular weight cannot be attributed solely to the incorporation of the microbiological treatment. Chemical treatment conditions, particularly reagent concentration, temperature, and exposure time, may also influence polymer chain length during processing [30].

These results are particularly relevant for the subsequent comparison of film properties. CM-Crb and Ch-Crb originated from the same biological source and exhibited similar MW values (~100 kDa), but differed in DDA and extraction approach. Conversely, CM-Shr and CM-Crb were obtained using the same hybrid chemical–microbiological extraction approach but differed in biological source and DDA. Together with the low residual ash contents, these differences provided a basis for evaluating the structural and functional properties of the resulting films under standardized film-forming conditions.

#### 3.1.2. Fourier Transform Infrared Spectroscopy (FTIR)

**Figure 1:**
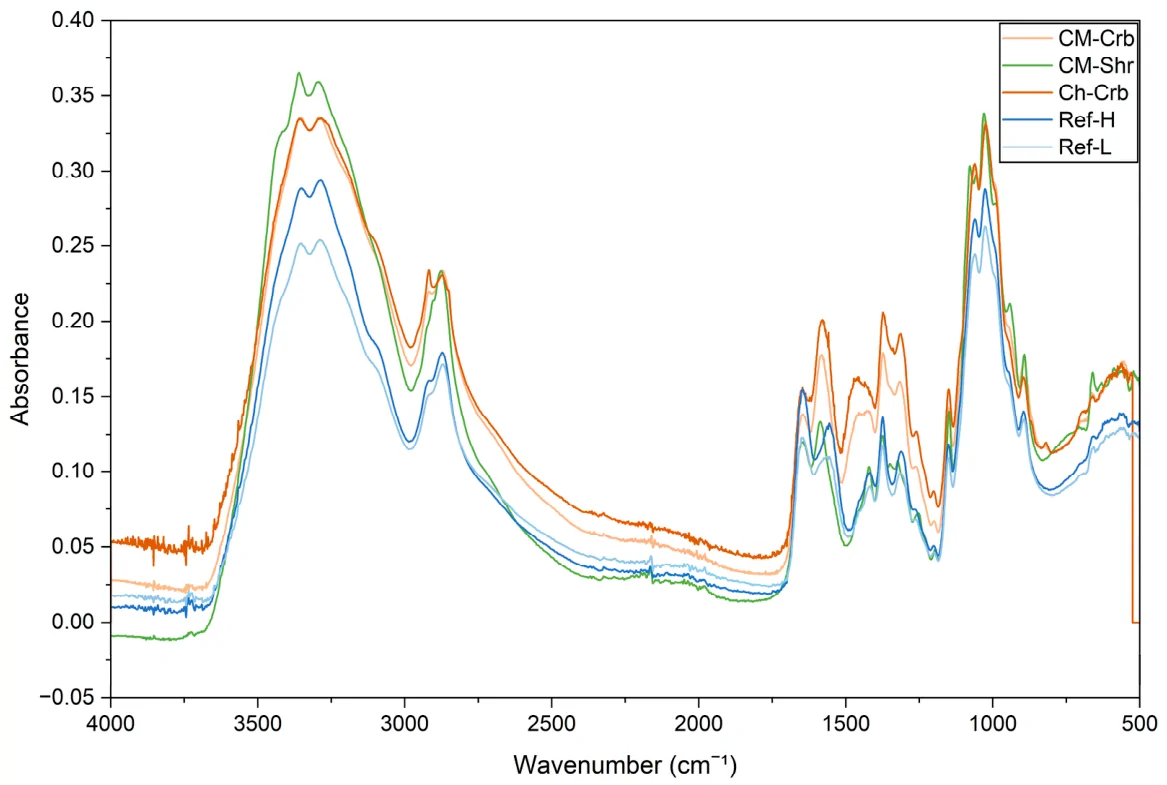
ATR-FTIR spectra of the starting chitosans CM-Crb, CM-Shr, Ch-Crb, Ref-H, and Ref-L recorded over the 4000–500 cm^−1^ range.

The ATR-FTIR spectra of the starting chitosans are shown in Figure 1. All samples exhibited the characteristic absorption bands of chitosan, although differences in band shape and position were observed. These spectral differences were examined together with the DDA and MW characteristics presented in Table 1 to assess variations in the chemical environments of the starting polymers. A broad absorption band was observed in the 3400–3200 cm^−1^ region, corresponding to overlapping O–H and N–H stretching vibrations involved in the hydrogen-bonding network of chitosan. Differences in the profile of this region among the samples suggest variations in the molecular environment and intermolecular interactions of the starting polymers. Such differences may contribute to their distinct hydration behavior; however, apparent water absorption of the resulting films is also influenced by other physicochemical and structural characteristics, including DDA, MW, chain organization, and film morphology. Because hydrogen bonding contributes to intermolecular association within chitosan networks, differences in this spectral region may also reflect variations in chain–chain interactions that can influence polymer organization during film formation.

Differences among the chitosans were particularly evident in the 1700–1200 cm^−1^ region. The band around 1650 cm^−1^ is commonly assigned to amide I, mainly associated with C=O stretching of residual N-acetyl groups, whereas the region around 1590–1570 cm^−1^ is primarily related to N–H bending of primary amino groups (–NH_2_). Amide II contributions may also occur around 1560–1550 cm^−1^ and overlap with the amino band [31,32,33,34]. Bands near 1420 and 1320 cm^−1^ have also been associated with the acetylation state of chitosan, with the latter containing contributions from amide III/C–N vibrations [31,33].

Among the experimental chitosans, CM-Crb exhibited a prominent band at 1582.0 cm^−1^, while CM-Shr showed a corresponding feature at 1586.6 cm^−1^. Ch-Crb presented a more complex profile, with features at 1579.9 and 1558.9 cm^−1^. The latter falls within the region where amide II contributions from residual N-acetyl groups may overlap with N–H bending of primary amino groups [32,33]. Therefore, the shoulders observed in this region, particularly for the crab-derived chitosans, may reflect differences in the relative contributions and local molecular environments of amino and residual N-acetyl functionalities rather than the presence of additional functional groups. These differences are potentially relevant to film functionality because the protonation and accessibility of amino groups are central to the electrostatic interactions generally associated with the antibacterial activity of chitosan [35,36]. The ATR-FTIR spectra did not provide sufficient evidence to identify specific residual impurities. Although ash content provided an estimate of residual inorganic material, other aspects of compositional purity were not independently quantified.

Differences were also observed at lower wavenumbers. CM-Crb, Ch-Crb, and CM-Shr showed maxima at 1428.9, 1458.4, and 1420.8 cm^−1^, respectively, while bands in the ~1320 cm^−1^ region occurred at 1316.4, 1312.8, and 1323.4 cm^−1^, respectively. These differences in band position and profile further indicate variations in the chemical environment of the functional groups among the experimental chitosans.

These spectral differences were considered together with the independently determined DDA values presented in Table 1. Thus, the quantitative differences in DDA were accompanied by measurable differences in the position and profile of bands associated with amino and residual N-acetyl functionalities, particularly in the ~1590–1550 and ~1320 cm^−1^ regions described above. Changes in chitosan deacetylation have been associated with variations in bands related to residual N-acetyl and amino functionalities. Brugnerotto et al. [31] demonstrated that the A_1320_/A_1420_ absorbance ratio is sensitive to the degree of acetylation, while Yarnpakdee et al. [34] reported decreasing signals near 1645 and 1320 cm^−1^ and increasing signals near 1590 and 1420 cm^−1^ with increasing deacetylation of crustacean-derived chitosan. Thus, the spectral differences observed among CM-Crb, Ch-Crb, and CM-Shr may be associated, at least in part, with differences in their acetylation characteristics. Nevertheless, differences in band position and profile should not be interpreted as a quantitative spectroscopic determination of acetylation. Because no quantitative FTIR absorbance-ratio analysis was performed, these spectral variations were interpreted qualitatively and were not used to estimate DDA. This distinction may also have functional implications. Lim et al. [20] directly demonstrated that both acetylation degree and MW affect intermolecular interactions in chitosan, with acetylation influencing intrinsic charge and chain flexibility and MW modifying the relative importance of chain mobility in these interactions. Therefore, differences in DDA may contribute to variations in intermolecular association and film behavior even when the chitosans have comparable MW values.

Notably, CM-Crb and Ch-Crb exhibited similar MW values (~100 kDa) but showed distinct spectral profiles, particularly in regions associated with amino, residual N-acetyl, and hydrogen-bonded functionalities. Accordingly, comparable average MW values do not necessarily imply equivalent molecular interactions or functional behavior [20]. Because CM-Crb and Ch-Crb originated from the same biological source but were obtained through hybrid chemical–microbiological and conventional chemical extraction, respectively, their different processing histories, together with their different DDA values, may have contributed to the observed spectral differences. Extraction conditions may alter polymer-chain characteristics and the extent of deacetylation, providing plausible pathways through which processing history could affect the subsequent properties of the films [11,12,13].

Furthermore, CM-Crb and CM-Shr were obtained using the same hybrid approach but from different crustacean sources, indicating that the observed spectral variability cannot be attributed exclusively to biological source or extraction route. Taken together, the FTIR and DDA results suggest that differences in the molecular environments of amino and residual N-acetyl groups, as well as hydrogen-bonding interactions, may contribute to the distinct functional responses of the films discussed in the following sections. However, the individual contributions of these characteristics cannot be isolated from the present experimental design.

### 3.2. Thermogravimetric Analysis

**Figure 2:**
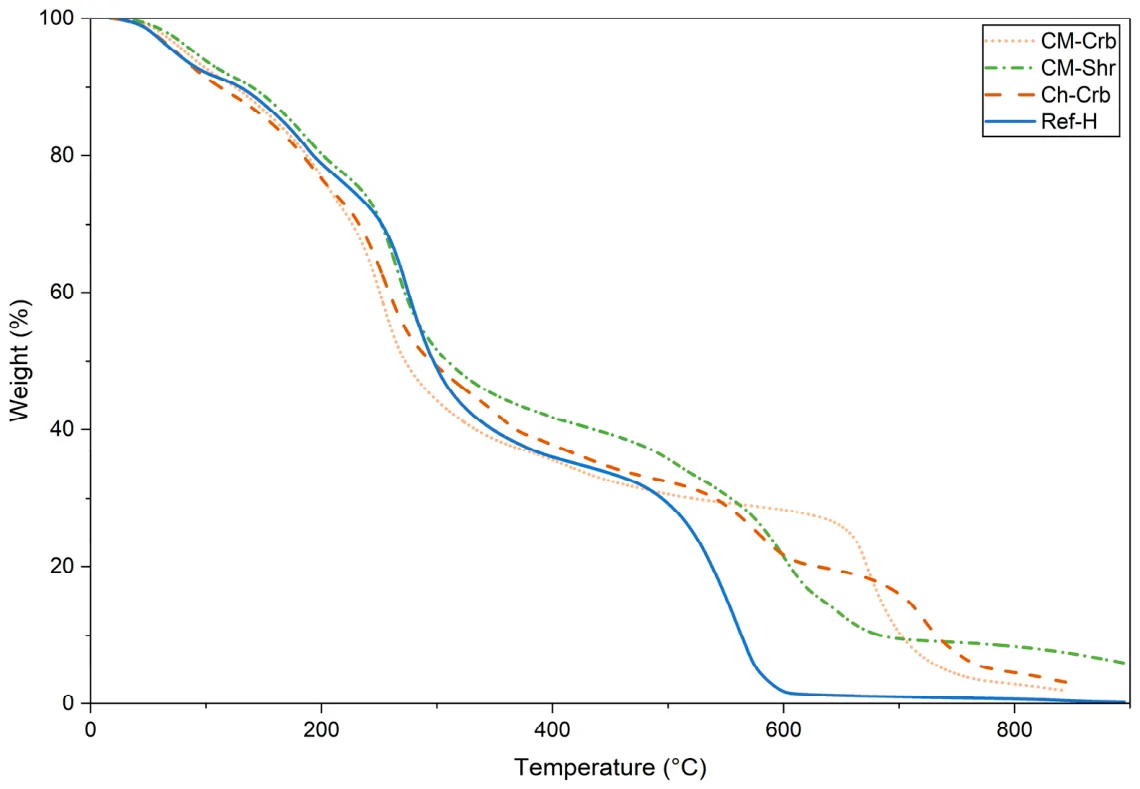
Thermogravimetric curves of chitosan films prepared from CM-Crb, CM-Shr, Ch-Crb, and Ref-H using film-forming solutions containing 1.5% (*w*/*v*) chitosan. All films were plasticized with glycerol at 30% (*w*/*w*, based on chitosan dry weight).

The thermal degradation behavior of the chitosan films was evaluated by TGA. The thermogravimetric (TG) curves of the prepared chitosan films are shown in Figure 2, while Table 2 summarizes the degradation temperatures of the main thermal events and their associated mass losses (Δw). Four main thermal events were identified in the films. Notably, the initial mass losses (Δw, 8.5–10.7%) recorded for the films in Table 2 were higher than the moisture contents determined for the corresponding starting chitosan powders in Table 1 (6.6–6.9%). This difference may be associated with the distinct sample compositions and analytical conditions, as Table 1 refers to unplasticized chitosan powders, whereas Table 2 corresponds to solvent-cast films containing 30% (*w*/*w*, based on chitosan dry weight) glycerol. The hygroscopic nature of glycerol may contribute to water retention within the film matrix.

The first thermal event occurred between 78.2 and 86.4 °C, with mass losses ranging from 8.5% to 10.7%. This low-temperature mass loss was attributed mainly to the evaporation of moisture and physically adsorbed water retained within the hydrophilic chitosan matrix. Corazzari et al. [37] reported that water associated with chitosan is released during the initial stage of thermal analysis, preceding the main degradation of the polysaccharide structure. The relatively narrow temperature interval observed among the films for this event suggests comparable initial moisture-release behavior, although differences in mass loss may reflect variations in the amount and strength of water associated with the polymer network.

A second thermal event was observed between 187.5 and 198.3 °C, corresponding to mass losses of 13.4–18.1%. Because the films contained glycerol and were analyzed as multicomponent polymeric matrices, this region was considered an intermediate thermal event rather than being assigned exclusively to bound water. Thermal changes in this region may involve the release of more strongly retained volatile components together with the onset of degradation processes within the plasticized chitosan matrix. Previous studies have reported variation in the thermal behavior of chitosan and chitosan-based films as a function of polymer characteristics and film composition [37,38,39,40]. In this context, differences in hydrogen bonding and polymer–glycerol interactions may modify the energy required for the release of associated water and volatile species and for the initiation of thermal decomposition. Accordingly, this intermediate event is more appropriately interpreted as the result of overlapping processes within the hydrated and plasticized polymer network rather than as a single-component transition.

The main degradation stage occurred between 252.8 and 272.6 °C, with mass losses ranging from 36.5% to 39.9%. This region was associated primarily with thermal decomposition of the chitosan-based polymeric matrix, involving degradation of the polysaccharide backbone and overlapping contributions from the plasticized film components [37,38]. Among the films, Ref-H exhibited the highest Td for this stage (272.6 °C), followed by CM-Shr (264.1 °C), CM-Crb (253.0 °C), and Ch-Crb (252.8 °C). The nearly 20 °C difference between Ref-H and Ch-Crb indicates that the polymer matrices did not exhibit equivalent resistance to thermal decomposition despite being prepared with the same nominal chitosan and glycerol proportions. Differences in MW, DDA, chain organization, and intermolecular interactions of the starting chitosans provide plausible contributions to this behavior, since MW and acetylation characteristics have been shown to influence chitosan chain mobility and intermolecular interactions [20]. These structural differences may, in turn, contribute to the distinct thermal responses observed among the films.

A fourth degradation region was observed at substantially higher temperatures and was associated with oxidative decomposition of the residual fraction remaining after the main degradation of the polymeric matrix. CM-Crb exhibited a Td of 673.6 °C with a mass loss of 26.0%, whereas CM-Shr and Ref-H showed Td values of 597.7 and 570.4 °C, with corresponding mass losses of 29.9% and 31.9%, respectively. Ch-Crb exhibited a Td of 580.0 °C with a corresponding mass loss of 15.2%. Because the analyses were performed under an air atmosphere and the chemical composition of the residual fraction was not independently characterized, these high-temperature events cannot be unequivocally assigned to a specific inorganic component or carbonaceous structure. This conservative interpretation is particularly appropriate because the thermal degradation of chitosan under oxidative conditions can involve multiple overlapping degradation processes [38]. The markedly higher temperature of this event for CM-Crb may therefore reflect differences in the nature or thermal stability of the residual material formed during the preceding degradation stages.

The multistep thermal profiles observed here are consistent with the complex degradation behavior reported for chitosan under oxidative conditions [37,38]. Moussout et al. [38] showed that chitosans with different degrees of deacetylation can exhibit different thermal responses under an air atmosphere, indicating that polymer characteristics can modify the position and progression of degradation events. Importantly, their results showed that the relationship between DDA and thermal stability is not necessarily linear; under oxidative conditions, the least deacetylated chitosan evaluated exhibited the highest thermal stability [38]. This supports considering DDA as a contributor to thermal behavior rather than assuming that a higher DDA necessarily produces a higher degradation temperature.

In addition, the conditions used for chitosan production can influence DDA and MW, and deacetylation under concentrated alkaline conditions, particularly at elevated temperatures or with prolonged or successive treatments, can promote polymer-chain depolymerization [11]. Changes in MW resulting from processing may, in turn, alter chain entanglement and intermolecular organization, while differences in acetylation modify the relative proportions of glucosamine and N-acetylglucosamine units participating in the polymer network. Thermal studies performed with chitosans of systematically varied DDA have confirmed that acetylation/deacetylation modifies the thermal decomposition behavior of chitosan, including its thermal stability and the composition of the products evolved during degradation [41].

These considerations are relevant because the starting chitosans evaluated here differed in both DDA and MW. Despite being prepared under standardized film-forming conditions, the films exhibited distinct thermal responses. The comparison between CM-Crb and Ch-Crb is particularly informative: despite their similar MW values (~100 kDa) and common crab origin, their main degradation temperatures were nearly identical, whereas CM-Shr and especially the higher-MW Ref-H exhibited higher main degradation temperatures. This pattern is compatible with a contribution of MW to thermal behavior. More broadly, the results suggest that the physicochemical characteristics of the starting chitosans collectively contributed to the thermal behavior of the resulting films.

### 3.3. Scanning Electron Microscopy (SEM)

**Figure 3:**
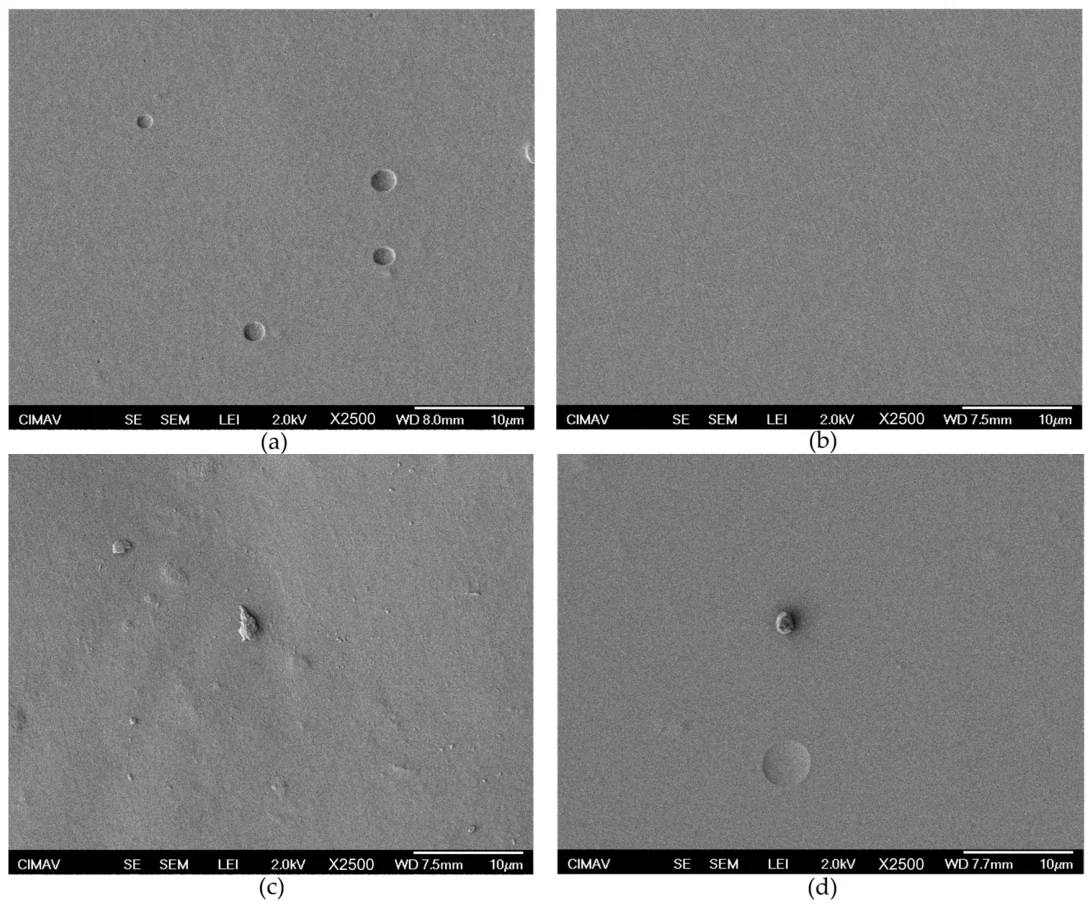
SEM micrographs of the top surface morphology of CM-Crb (**a**), CM-Shr (**b**), Ch-Crb (**c**), and Ref-H (**d**) chitosan films prepared from film-forming solutions containing 1.5% (*w*/*v*) chitosan at 2500× magnification. All films were plasticized with glycerol at 30% (*w*/*w*, based on chitosan dry weight).

**Figure 4:**
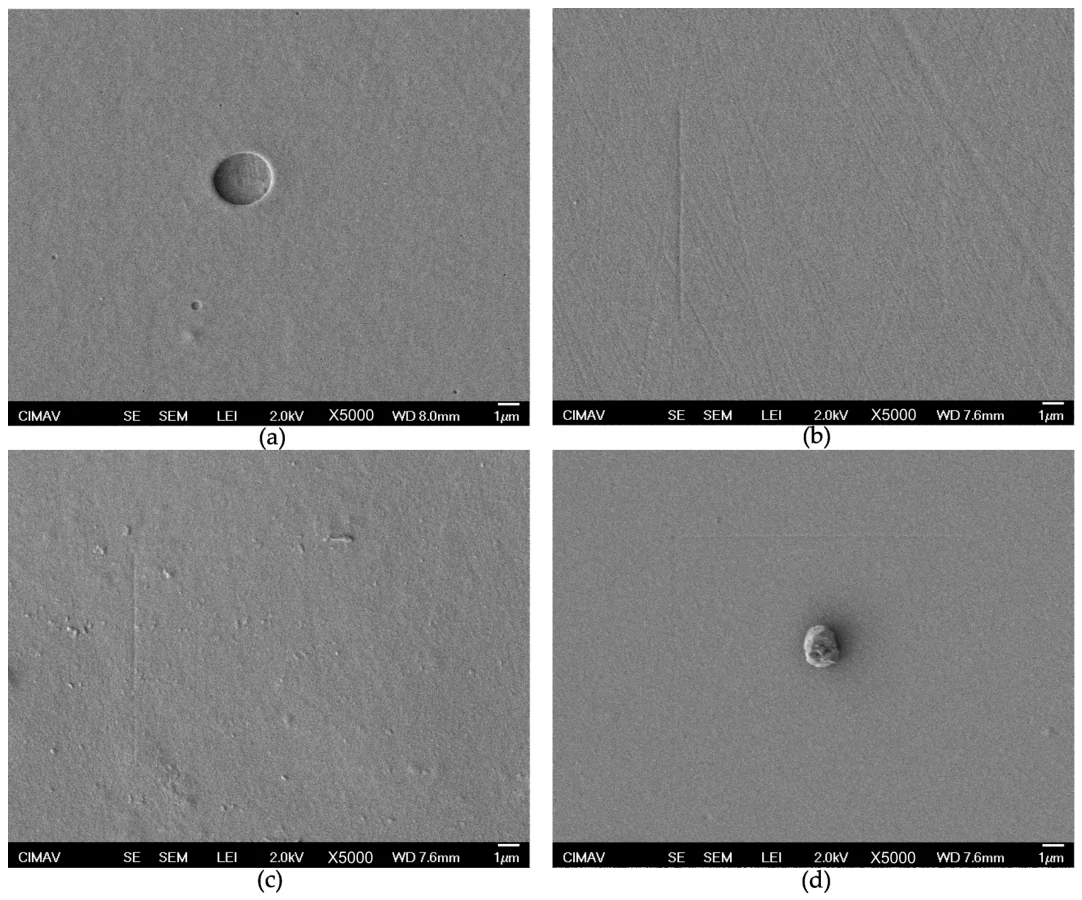
SEM micrographs of the top surface morphology of CM-Crb (**a**), CM-Shr (**b**), Ch-Crb (**c**), and Ref-H (**d**) chitosan films prepared from film-forming solutions containing 1.5% (*w*/*v*) chitosan at 5000× magnification. All films were plasticized with glycerol at 30% (*w*/*w*, based on chitosan dry weight).

Scanning electron microscopy was used to examine the surface morphology of the chitosan films. The SEM images revealed differences in surface homogeneity, apparent porosity, and the presence of particulate or aggregate-like features among the films (Figure 3 and Figure 4). CM-Shr exhibited the most homogeneous and compact surface, with no visible cracks and relatively low apparent porosity at the evaluated magnifications. In contrast, CM-Crb displayed a more heterogeneous and porous surface, with apparent pore diameters of approximately 0.5–2.5 μm. Ch-Crb showed surface-associated particulate or aggregate-like structures of approximately 1–2 μm, whereas Ref-H exhibited larger surface features of approximately 1–3 μm together with pores reaching approximately 3.5 μm estimated from micrographs.

Differences in chitosan molecular characteristics can affect intermolecular interactions and polymer-matrix organization during film formation. Zhang et al. [42], who compared chitosan films prepared from polymers with molecular weights ranging from 30 to 300 kDa, reported molecular-weight-dependent differences in film-forming behavior, physicochemical properties, and microstructure. In the present study, the heterogeneous surface of Ref-H may therefore be associated, at least in part, with its higher molecular-weight range (310–375 kDa). Notably, CM-Crb and Ch-Crb originated from the same biological source and had similar MW values (~100 kDa), yet they exhibited distinct surface morphologies, with CM-Crb showing more evident apparent porosity and Ch-Crb displaying predominantly particulate or aggregate-like surface features. However, CM-Crb and Ch-Crb also differed in DDA and potentially in other physicochemical characteristics not quantified in the present study; therefore, their distinct surface morphologies cannot be attributed specifically to the extraction approach. CM-Crb exhibited a lower DDA (75.1 ± 2.3%) than Ch-Crb (80.2 ± 2.1%), corresponding to a greater relative proportion of N-acetylglucosamine units. Lim et al. [20] demonstrated that the acetylation degree influences chitosan intermolecular interactions by modifying intrinsic charge and chain flexibility, while MW determines the relative contribution of chain mobility to these interactions. Differences in acetylation state and intermolecular organization therefore provide plausible contributing factors to the distinct surface morphologies observed for CM-Crb and Ch-Crb.

The SEM observations were also compared with the hydration behavior of the films. CM-Shr combined a relatively compact surface with lower water uptake than Ref-H, whereas CM-Crb exhibited the most evident apparent porosity without correspondingly high apparent water absorption. At 15 min, for example, CM-Crb showed an apparent water absorption of 34.5 ± 1.2%, compared with 51.8 ± 1.9% for Ref-H. This behavior suggests that water uptake was not governed simply by the visible surface features but also by the molecular organization of the chitosan matrix. Previous studies have shown that the DDA affects water sorption in chitosan films and that this response is associated with polymer–water interactions, chain stiffness, and crystalline organization [43]. Therefore, the greater apparent surface porosity of CM-Crb did not necessarily result in greater apparent water absorption because water uptake also depends on the molecular organization and hydration behavior of the polymer network. Because only film surfaces were examined, these micrographs should not be interpreted as evidence of internal or through-thickness porosity.

Overall, the SEM analysis showed that the standardized film-forming procedure produced distinct surface morphologies depending on the chitosan used. CM-Shr formed the most homogeneous and compact surface, whereas CM-Crb, Ch-Crb, and Ref-H showed more evident porous, particulate, or heterogeneous features. The combined SEM, MW, and DDA results therefore suggest that the observed surface morphologies arose from differences in molecular characteristics that affected intermolecular association and polymer organization during film formation [20,42].

### 3.4. Apparent Water Absorption Behavior

**Figure 5:**
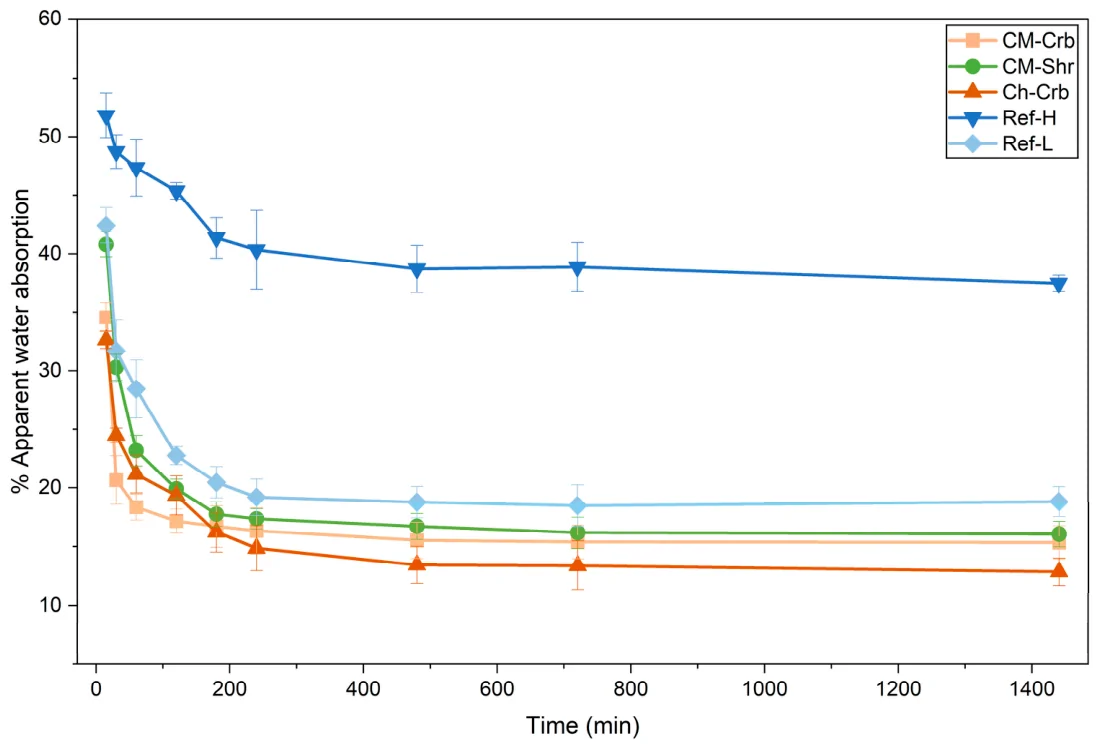
Apparent water absorption profiles of chitosan films prepared from film-forming solutions containing 1.5% (*w*/*v*) chitosan during immersion in phosphate buffer (pH 7.4) for 1440 min. Values are expressed as mean ± standard deviation (*n* = 4). The same film specimens were monitored throughout the immersion period. All films were plasticized with glycerol at 30% (*w*/*w*, based on chitosan dry weight).

Water absorption is an important functional property of chitosan films because it reflects the interaction between the hydrophilic polymer matrix and the surrounding aqueous environment. Figure 5 shows the apparent water absorption profiles of the 1.5% (*w*/*v*) chitosan films during immersion in phosphate buffer (pH 7.4) for 15–1440 min. The highest apparent absorption values were recorded at 15 min, corresponding to the first measurement. At this time, film type had a significant effect on apparent water absorption (one-way ANOVA, *F* = 125.23, *p* < 0.001). The assumption of homogeneity of variances was satisfied (Levene’s test, *p* > 0.05). Tukey’s HSD test separated the films into three homogeneous groups. Ref-H, prepared from high-molecular-weight commercial chitosan, exhibited the highest apparent water absorption (51.8 ± 1.9%; group A). Ref-L (42.4 ± 1.5%) and CM-Shr (40.7 ± 1.0%) formed a second homogeneous group (B), whereas CM-Crb (34.5 ± 1.2%) and Ch-Crb (32.6 ± 0.7%) showed the lowest values and formed a third homogeneous group (C).

Among the experimentally produced chitosans, CM-Shr and CM-Crb, which were obtained using the same hybrid chemical–microbiological extraction approach but from different biological sources, differed significantly in their 15-min apparent water absorption (40.7 ± 1.0% and 34.5 ± 1.2%, respectively; *p* < 0.05). In contrast, CM-Crb and Ch-Crb, both derived from crab but obtained through different extraction approaches, showed statistically comparable values (34.5 ± 1.2% and 32.6 ± 0.7%, respectively; *p* > 0.05). The similarity between the two crab-derived films suggests that their polymer matrices developed comparable initial hydration capacities despite differences in extraction approach and DDA, whereas the higher absorption of CM-Shr may reflect differences in the organization of the shrimp-derived chitosan network under the same nominal film-forming conditions.

Following the initial hydration stage, the apparent water absorption values progressively decreased with increasing immersion time, although the magnitude of this decrease varied among the films. At 1440 min, apparent water absorption was 37.4 ± 0.6% for Ref-H, 18.8 ± 1.2% for Ref-L, 16.0 ± 1.0% for CM-Shr, 15.3 ± 1.4% for CM-Crb, and 12.8 ± 1.1% for Ch-Crb. The rapid initial uptake is consistent with the hydrophilic character of chitosan, whose hydroxyl and amino groups promote interactions with water through hydrogen bonding. Hydration can also modify chitosan-chain mobility and molecular organization; water-induced changes in hydrogen bonding, electrostatic interactions, and crystalline organization have been reported in chitosan films [44]. The subsequent progressive decrease in the calculated apparent water-absorption values during prolonged immersion may be associated with changes in the hydrated film matrix and potential loss of soluble components. A comparable temporal trend was reported by Schmidt et al. [45] for glycerol-plasticized chitosan films, which exhibited rapid initial water uptake followed by a progressive decrease in relative wet mass during prolonged immersion. Because the chitosan films were prepared in acetic acid and were neither chemically cross-linked nor converted to the free-base form, partial solubilization during immersion is plausible. However, although the films remained macroscopically intact, mass loss was not independently quantified, and the relative contributions of water uptake and material loss cannot be distinguished.

Notably, Ref-H, the high-molecular-weight commercial reference, exhibited the greatest and most persistent apparent water uptake throughout the experiment. Previous studies have shown that MW and plasticizer content can influence water-related properties of chitosan films, although the specific response depends on film composition, experimental conditions, and the property evaluated [21,46,47]. Sutharsan et al. [21], for example, reported increased swelling ability with increasing chitosan MW. The significantly higher initial apparent water absorption of Ref-H (51.8 ± 1.9%) is therefore consistent with a possible contribution of its higher MW (310–375 kDa) to the hydration behavior of the film.

However, comparison among the experimentally produced chitosans revealed different hydration responses despite their relatively similar estimated viscosity-average molecular weights. CM-Shr and CM-Crb had comparable MW values but exhibited significantly different apparent water absorption at 15 min (40.7 ± 1.0% and 34.5 ± 1.2%, respectively). Their DDA values also differed (80.8 ± 0.3% for CM-Shr and 75.1 ± 2.3% for CM-Crb), indicating different relative proportions of glucosamine and N-acetylglucosamine units. In contrast, CM-Crb and Ch-Crb had similar estimated MW values (~100 kDa) and statistically comparable apparent water absorption at 15 min (34.5 ± 1.2% and 32.6 ± 0.7%, respectively), despite differences in DDA and processing history. Gámiz-González et al. [43] demonstrated that N-acetylation can substantially affect water sorption in chitosan films and related this behavior particularly to changes in crystalline organization during swelling. These findings highlight the importance of considering the combined physicochemical characteristics of the starting chitosans when interpreting film hydration behavior, although the individual contributions of MW, DDA, and molecular organization cannot be resolved from the present experimental design.

### 3.5. Antibacterial Properties

**Figure 6:**
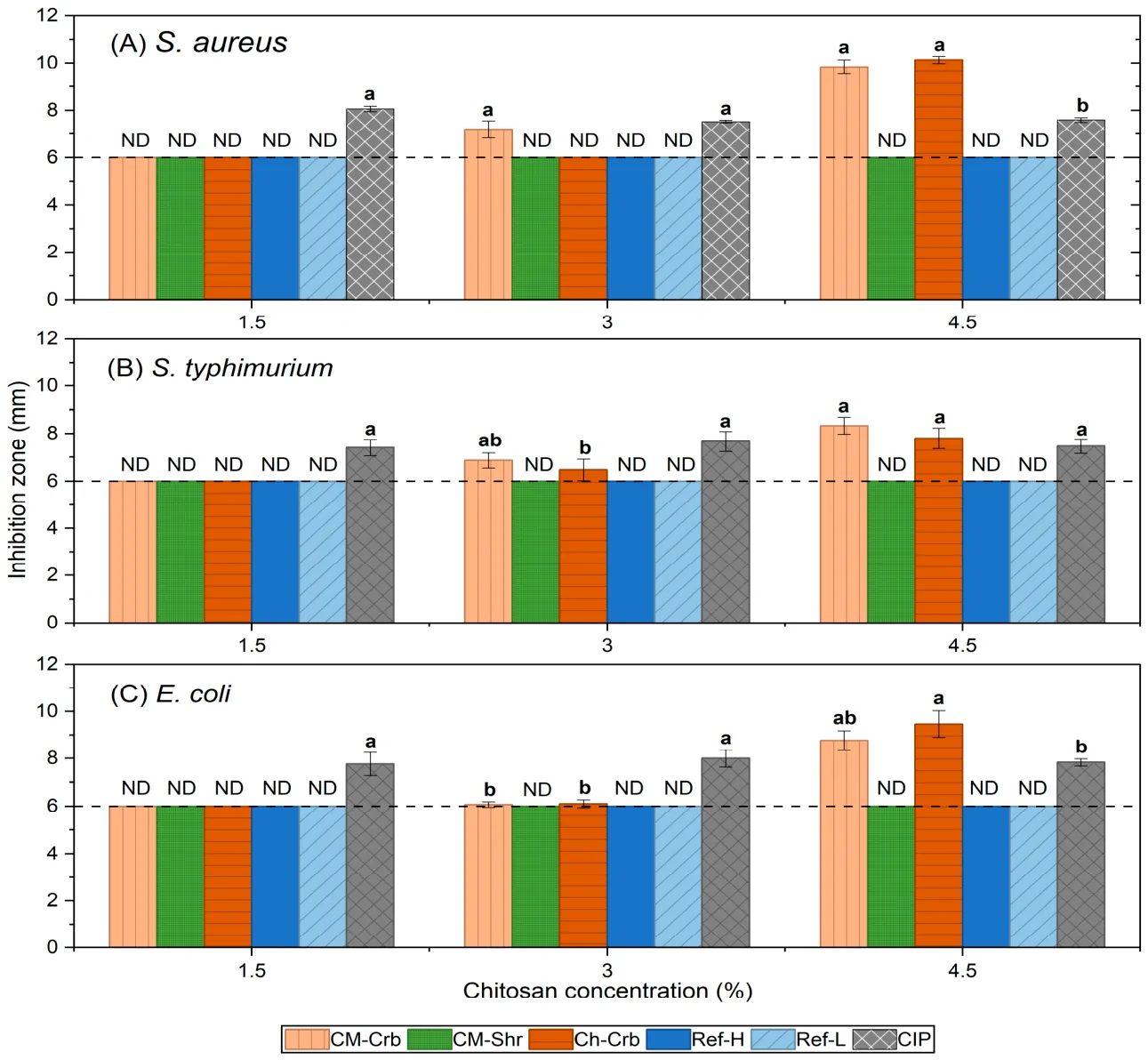
Antibacterial activity of chitosan films prepared from film-forming solutions containing 1.5%, 3.0%, and 4.5% (*w*/*v*) chitosan against (**A**) *S. aureus* ATCC 29213, (**B**) *S. Typhimurium* ATCC 14028, and (**C**) *E. coli* ATCC 25922, as determined by the agar disk-diffusion assay. Films contained 30% (*w*/*w*, based on chitosan dry weight) glycerol. CIP was included as the positive control. Results are expressed as total inhibition diameter (mean ± SD). The dashed line indicates the 6 mm film-disk diameter. ND indicates no detectable inhibition beyond the disk diameter (6.0 ± 0.0 mm). Different lowercase letters indicate significant differences among treatments exhibiting detectable inhibition within each concentration and bacterial strain (Tukey’s HSD, *p* < 0.05). Treatments classified as ND were excluded from these multiple comparisons.

**Figure 7:**
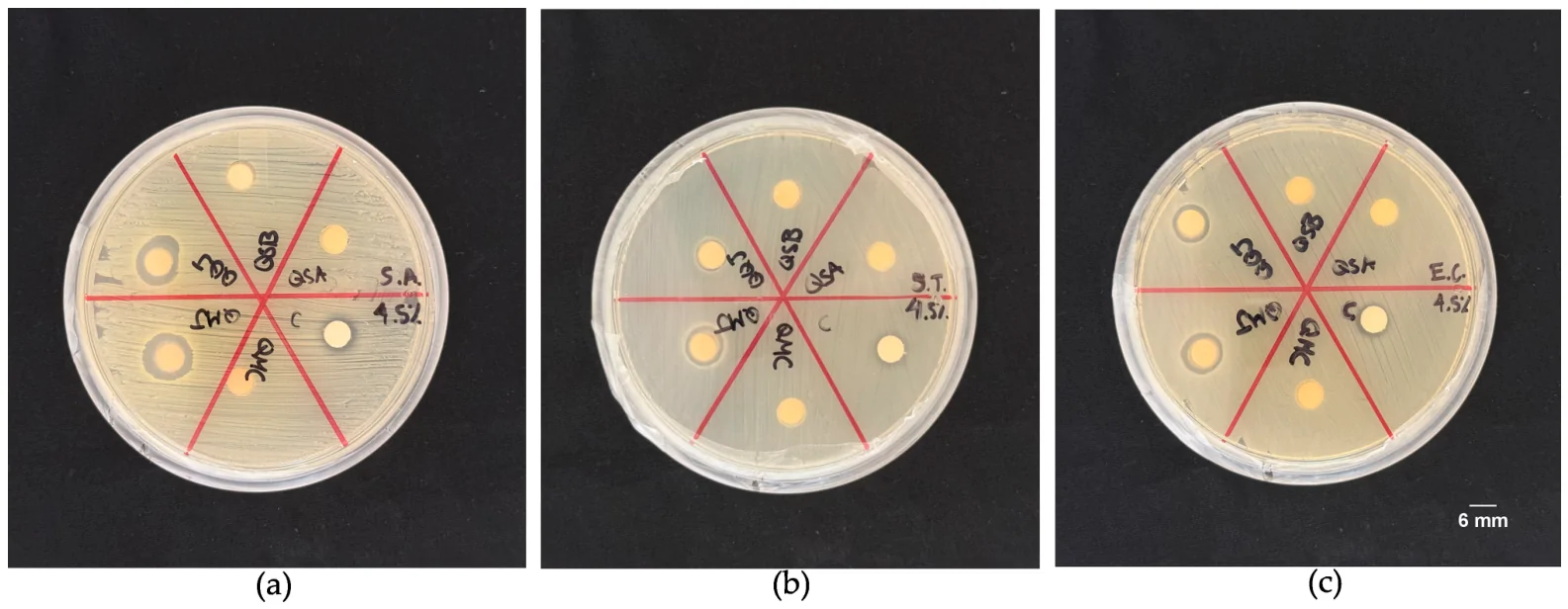
Representative photographs of the agar disk-diffusion assay of chitosan films prepared from film-forming solutions containing 4.5% (*w*/*v*) chitosan against (**a**) *S. aureus* ATCC 29213, (**b**) *S. Typhimurium* ATCC 14028, and (**c**) *E. coli* ATCC 25922. Ciprofloxacin was used as the positive control. Film disks were 6 mm in diameter. A calibrated 6 mm scale bar is included in panel (**c**) and applies to all panels, which are presented at the same scale. Note: The original laboratory markings on the Petri dishes correspond to CM-Crb (QMJ), Ch-Crb (QQJ), CM-Shr (QMC), Ref-H (QSA), Ref-L (QSB), and CIP (C).

The antibacterial activity of the chitosan films was evaluated by the agar disk-diffusion assay against *Staphylococcus aureus* ATCC 29213, *Salmonella Typhimurium* ATCC 14028, and *Escherichia coli* ATCC 25922 using films prepared from film-forming solutions containing 1.5%, 3.0%, and 4.5% (*w*/*v*) chitosan (Figure 6). Antibacterial activity was assessed as the total inhibition diameter, including the 6 mm film disk and any surrounding clear zone (Figure 7). CIP, included as the positive control, produced inhibition zones against all three bacterial strains, confirming their susceptibility and the ability of the agar diffusion assay to detect antibacterial activity.

For films prepared from 1.5% (*w*/*v*) chitosan solutions, none of the five formulations produced detectable inhibition beyond the 6 mm film-disk diameter against any of the three bacterial strains. Similar findings have been reported for chitosan-based materials evaluated by diffusion-based methods, in which no detectable inhibition zones were observed under the tested conditions [48]. At a chitosan concentration of 3.0% (*w*/*v*) in the film-forming solution, inhibition beyond the disk diameter was observed for CM-Crb against all three bacterial strains and for Ch-Crb against *S. Typhimurium* and *E. coli*. At a chitosan concentration of 4.5% (*w*/*v*) in the film-forming solution, both crab-derived formulations (CM-Crb and Ch-Crb) exhibited detectable inhibition against all three strains. In contrast, CM-Shr, Ref-H, and Ref-L remained at the 6 mm disk diameter across all concentrations and bacterial strains evaluated. Formulations consistently exhibiting a total diameter of 6.0 ± 0.0 mm were classified as showing no detectable inhibition (ND) and reported descriptively.

To further evaluate the antibacterial responses, separate two-way ANOVAs were performed for each bacterial strain using the subset comprising CM-Crb, Ch-Crb, and CIP at film-forming solution chitosan concentrations of 3.0% and 4.5% (*w*/*v*). The Shapiro–Wilk test did not indicate significant departures from residual normality for *S. aureus* (*p* = 0.4173), *S. Typhimurium* (*p* = 0.0659), or *E. coli* (*p* = 0.6244). Similarly, Levene’s test did not detect significant heterogeneity of variances (*p* = 0.3290, 0.9965, and 0.6481, respectively). Significant treatment × concentration interactions were observed for *S. aureus* (F(2,12) = 146.4, *p* < 0.0001), *S. Typhimurium* (F(2,12) = 8.7, *p* = 0.0046), and *E. coli* (F(2,12) = 45.7, *p* < 0.0001). These findings indicate that, within the analyzed subset, the relationship between treatment and inhibition diameter differed between the two concentrations evaluated.

For *S. aureus*, CM-Crb produced a mean inhibition diameter of 7.2 ± 0.4 mm at a chitosan concentration of 3.0% (*w*/*v*) in the film-forming solution, whereas Ch-Crb showed no detectable inhibition beyond the disk diameter. At a chitosan concentration of 4.5% (*w*/*v*) in the film-forming solution, CM-Crb and Ch-Crb produced inhibition diameters of 9.8 ± 0.3 and 10.1 ± 0.2 mm, respectively, without a significant difference between these two formulations according to Tukey’s HSD test (*p* > 0.05). Both crab-derived formulations exhibited significantly greater inhibition diameters than CIP at this concentration (*p* < 0.05), as indicated in Figure 6.

Against *S. Typhimurium*, CM-Crb and Ch-Crb exhibited inhibition diameters of 6.9 ± 0.3 and 6.5 ± 0.5 mm, respectively, at a chitosan concentration of 3.0% (*w*/*v*) in the film-forming solution. At this concentration, the Tukey HSD comparisons indicated overlapping statistical groups for CM-Crb, whereas Ch-Crb differed significantly from CIP. Increasing the film-forming solution chitosan concentration to 4.5% (*w*/*v*) resulted in inhibition diameters of 8.3 ± 0.4 mm for CM-Crb and 7.8 ± 0.4 mm for Ch-Crb. These results indicate a greater inhibition response at the higher concentration for both crab-derived formulations.

For *E. coli*, inhibition at a chitosan concentration of 3.0% (*w*/*v*) in the film-forming solution was limited, with mean inhibition zones of 6.1 ± 0.1 and 6.1 ± 0.2 mm for CM-Crb and Ch-Crb, respectively. Although these values indicated slight inhibition beyond the disk diameter in some replicates, both formulations exhibited significantly smaller inhibition diameters than CIP (Tukey’s HSD, *p* < 0.05). At a chitosan concentration of 4.5% (*w*/*v*) in the film-forming solution, the inhibition diameters increased to 8.8 ± 0.4 mm for CM-Crb and 9.5 ± 0.6 mm for Ch-Crb. The Tukey HSD comparisons indicated that Ch-Crb exhibited significantly greater inhibition than CIP, whereas CM-Crb belonged to an overlapping statistical group, as shown in Figure 6.

A consistent pattern nevertheless emerged across the three microorganisms: detectable inhibition beyond the 6 mm film-disk diameter was predominantly associated with the crab-derived chitosans (CM-Crb and Ch-Crb), whereas CM-Shr, Ref-H, and Ref-L showed no detectable inhibition beyond this diameter under the tested conditions. For both crab-derived films, mean inhibition diameters were greater when the chitosan concentration in the film-forming solution increased from 3.0% to 4.5% (*w*/*v*), consistent with the concentration dependence reported for chitosan antimicrobial activity [35]. The different strain-dependent patterns observed for CM-Crb and Ch-Crb suggest that, under the experimental conditions evaluated, the observed antibacterial response was not determined solely by Gram classification. At a chitosan concentration of 4.5% (*w*/*v*) in the film-forming solution, CM-Crb produced a numerically greater inhibition diameter against the Gram-positive S. aureus than against the two Gram-negative strains. However, this descriptive pattern was not reproduced by Ch-Crb, for which the mean inhibition diameter against *E. coli* was closer to that observed against *S. aureus* than to that observed against *S. Typhimurium*.

Chitosan antibacterial activity is primarily associated with electrostatic interactions between protonated amino groups (–NH_3_^+^) and negatively charged components of the bacterial cell envelope, and the magnitude of this interaction depends on both chitosan characteristics and the target microorganism [35,36,49]. Thus, differences in cell-envelope composition may contribute to the strain-dependent responses observed here, but the resulting antibacterial response may also depend on the physicochemical characteristics of the chitosan matrix.

The absence of detectable inhibition for Ref-L, despite its MW range (50–190 kDa) overlapping the estimated viscosity-average molecular-weight values of the experimentally produced chitosans (~100–110 kDa), contrasts with the detectable inhibition observed for CM-Crb and Ch-Crb. Similarly, CM-Shr and CM-Crb exhibited relatively close estimated MW values but differed in their antibacterial responses. These comparisons demonstrate that materials with overlapping or relatively similar reported molecular-weight values exhibited different antibacterial behavior under the tested conditions. However, the chitosans also differed in DDA, biological source, processing history, and other physicochemical characteristics, preventing the independent contribution of MW from being established.

Because molecular weight distribution (MWD) and the relative abundance of low-molecular-weight or oligomeric fractions were not characterized, their potential contribution to the observed differences cannot be excluded, particularly considering the complex relationship between chitosan molecular characteristics and antimicrobial performance reported by Másson [49]. Likewise, the antibacterial response did not follow a simple relationship with DDA. Ch-Crb (80.2 ± 2.1%) and CM-Shr (80.8 ± 0.3%) had numerically similar DDA values, yet inhibition beyond the film-disk diameter was detected for Ch-Crb, whereas CM-Shr showed no detectable inhibition under the tested conditions. Moreover, CM-Crb exhibited the lowest DDA (75.1 ± 2.3%) but also showed antibacterial activity.

However, DDA alone does not directly indicate the number of protonated amino groups under the film-forming conditions used here. Because both chitosan concentration and acetic acid concentration were maintained constant across formulations, differences in DDA resulted in different numbers of available amino groups and, consequently, different acid-to-amino-group ratios. Therefore, the extent of amino-group protonation cannot be assumed to vary proportionally with DDA. Since the actual degree of protonation was not experimentally determined, its contribution to the observed antibacterial differences cannot be established from the present data. Together with the FTIR differences in amino/N-acetyl and hydrogen-bonding regions, these results suggest that DDA alone was insufficient to explain the observed antibacterial behavior and that MW, acetylation characteristics, and intermolecular organization may have collectively contributed to the observed response [20].

Furthermore, CM-Crb and CM-Shr were obtained using the same hybrid chemical–microbiological extraction approach but differed in biological source, whereas CM-Crb and Ch-Crb shared the same crab origin but were obtained using different extraction approaches. Thus, the preferential inhibition observed for the two crab-derived chitosans represents a source-associated pattern; however, the factors underlying this pattern cannot be established from the present experimental design. Because agar diffusion assays depend on the availability and migration of active components into the surrounding medium, the inhibition diameters observed here should be interpreted as assay-dependent measures reflecting both antibacterial effects and the diffusion behavior of components released from the film matrix [48].

### 3.6. Mechanical Tests

The mechanical properties of the chitosan films are summarized in Table 3. Tensile strength differed significantly among formulations (ANOVA, *p* = 0.0198), ranging from 37.6 ± 6.6 MPa for Ch-Crb to 56.4 ± 7.2 MPa for Ref-H. CM-Crb and CM-Shr showed values of 52.1 ± 4.3 and 47.3 ± 4.6 MPa, respectively, whereas Ref-L reached 42.2 ± 6.1 MPa. Despite this numerical range, Tukey’s HSD test identified a significant pairwise difference only between Ref-H and Ch-Crb (*p* < 0.05); CM-Crb, CM-Shr, and Ref-L belonged to overlapping homogeneous groups.

Elongation at break also differed significantly among films (ANOVA, *p* = 0.0047) but followed a different pattern from TS. CM-Crb exhibited the highest EAB (3.7 ± 0.3%), significantly exceeding CM-Shr (2.3 ± 0.4%), Ch-Crb (2.5 ± 0.4%), and Ref-L (2.5 ± 0.2%). Ref-H showed an intermediate EAB of 3.2 ± 0.2% and did not differ significantly from either group. Notably, CM-Crb combined the highest deformation at failure with a relatively high TS (52.1 ± 4.3 MPa), indicating that its greater EAB was not accompanied by a marked reduction in tensile resistance under the conditions evaluated.

The comparison between the two crab-derived chitosans is particularly relevant because CM-Crb and Ch-Crb shared the same biological source and had similar estimated viscosity-average molecular weights (~100 kDa), but were obtained through different extraction approaches. Their EAB values differed significantly (3.7 ± 0.3 and 2.5 ± 0.4%, respectively), while CM-Crb also showed a numerically higher TS than Ch-Crb (52.1 ± 4.3 vs. 37.6 ± 6.6 MPa), although this pairwise difference was not significant according to Tukey’s test. Under standardized film-forming conditions, this comparison shows that two chitosans with similar estimated viscosity-average molecular weights exhibited significantly different deformation behavior. However, CM-Crb and Ch-Crb also differed in DDA and exhibited distinct ATR-FTIR profiles; therefore, the individual contributions of molecular weight, extraction approach, and acetylation characteristics to the observed mechanical differences cannot be established. Rather, their distinct mechanical responses may reflect differences in the overall physicochemical profiles of the starting chitosans, including acetylation state and intermolecular interactions, although the individual contributions of these characteristics cannot be resolved from the present experimental design. This interpretation is consistent with evidence that the degree of acetylation can modify chitosan chain flexibility and intermolecular interactions even at comparable MW [20].

Young’s modulus showed a significant overall effect among formulations (ANOVA, *p* = 0.0350), ranging from 2.2 ± 0.6 GPa for Ch-Crb to 3.5 ± 0.4 GPa for Ref-H. CM-Crb exhibited a similar numerical value (3.4 ± 0.3 GPa), followed by CM-Shr (2.9 ± 0.7 GPa) and Ref-L (2.2 ± 0.3 GPa). However, Tukey’s HSD test did not identify significant pairwise differences, with all five films remaining within the same homogeneous group. Therefore, although Ref-H and CM-Crb showed the highest numerical moduli, the pairwise analysis does not support greater stiffness of either film relative to a specific formulation.

All films contained the same glycerol proportion (30% *w*/*w* relative to chitosan). Bharti et al. [50] reported that glycerol incorporation decreased YM and increased deformation in chitosan films, consistent with its plasticizing effect. Since glycerol concentration was constant across the five formulations in the present study, differences in mechanical behavior cannot be attributed to differences in the amount of plasticizer added. Instead, the differences in EAB despite a constant glycerol proportion further support a contribution from the intrinsic physicochemical characteristics of the chitosans and their intermolecular organization.

SEM and tensile testing provide complementary but distinct information on the surface morphology and bulk mechanical behavior of the films, respectively. CM-Shr presented a comparatively uniform surface, CM-Crb exhibited more evident apparent surface porosity, and Ch-Crb displayed particulate and irregular surface features. In the tensile tests, CM-Crb maintained a relatively high TS and showed the highest EAB, whereas Ch-Crb showed the lowest TS. These morphological and mechanical observations are therefore considered independently rather than as evidence of a direct relationship between surface morphology and tensile behavior.

Taken together, the chitosan films exhibited distinct mechanical profiles rather than a common ranking across TS, EAB, and YM. These results indicate that mechanical performance was not governed by MW, glycerol content, or surface morphology individually, but was associated with the combined physicochemical characteristics and molecular organization of each chitosan film [20].

### 3.7. Optical Tests

The optical properties of the chitosan films were evaluated by measuring light transmission over the UV–visible region (200–800 nm). The main optical parameters are summarized in Table 4. Because a single measurement was obtained for each formulation (*n* = 1), the results are presented descriptively. Total light transmittance ranged from 84.8 to 89.8%, with the highest measured value observed for CM-Shr (89.8%), followed by CM-Crb (88.4%), Ref-H (87.0%), and Ch-Crb (84.8%).

At 340 nm, CM-Crb showed the highest measured UV blocking (35%), compared with 18% for CM-Shr and Ch-Crb and 17% for Ref-H. Thus, CM-Crb combined 88.4% total light transmittance with the highest measured UV attenuation among the evaluated films. The contrast between CM-Crb and Ch-Crb is noteworthy because both chitosans were crab-derived and had similar MW values (~100 kDa), yet CM-Crb showed 35% UV blocking at 340 nm compared with 18% for Ch-Crb. This difference despite comparable MW indicates that viscosity-average MW alone was insufficient to explain the observed difference in UV attenuation. Visual inspection revealed relatively uniform coloration within each film formulation. CM-Crb exhibited a faint brownish tint while remaining translucent, whereas the other films appeared relatively transparent.

Diffused-light values ranged from 4.4 to 6.3%, with the lowest measured value observed for CM-Crb (4.4%), followed by Ch-Crb (5.7%), Ref-H (6.2%), and CM-Shr (6.3%). Differences in light scattering among polymeric films may reflect variations in film organization and optical heterogeneity; however, the present measurements do not allow the factors responsible for these differences to be identified.

The relatively high light transmittance observed here is generally consistent with values reported for chitosan-based films. Liu et al. [29], for example, reported a light transmission of 91.2% for an optimized high-molecular-weight chitosan film formulation. Direct quantitative comparison, however, should be made cautiously because optical measurements can be affected by polymer characteristics, film composition, thickness, and measurement conditions.

Optical transparency and UV attenuation are potentially complementary properties for food-packaging materials, because transparency facilitates product visualization, whereas UV attenuation may reduce the exposure of packaged foods to radiation associated with photooxidative deterioration. Enhanced UV protection has also been reported for functionalized chitosan films containing UV-active components [51,52]. In the present study, no exogenous UV-blocking agents were incorporated into the films, making the differences observed among the starting chitosans particularly relevant.

Overall, CM-Crb showed a distinctive optical profile, combining high total light transmittance, low diffuse-light transmission, and the highest measured UV blocking at 340 nm. This combination suggests potential for food-packaging applications in which product visibility and partial attenuation of UV radiation are desirable. Nevertheless, replicated optical measurements and further studies using actual food systems would be required to confirm the reproducibility of these optical characteristics and determine whether the observed UV attenuation translates into improved protection against photooxidative deterioration during storage.

An important limitation of the present study is that the experimental design was not factorial and therefore does not allow the independent effects of biological source, extraction approach, MW, and DDA to be separated. Although all films were prepared and evaluated under standardized conditions, these characteristics varied simultaneously among some of the starting chitosans. The physicochemical characterization of the experimentally produced starting chitosans included DDA, MW, moisture, ash content, total nitrogen content, and ATR-FTIR analysis; however, other potentially relevant characteristics, including crystallinity, molecular-weight distribution, and a broader assessment of compositional purity, were not experimentally determined. Consequently, the observed differences in film properties should be interpreted as being associated with the overall physicochemical profiles of the evaluated chitosans rather than attributed to any single material factor. Further studies using systematically controlled and more comprehensively characterized chitosans would be required to determine the individual contributions and possible interactions of biological source, extraction approach, MW, DDA, and other physicochemical characteristics to film performance.

## 4. Conclusions

This study showed that chitosans differing in biological source, extraction approach, DDA, and MW produced films with distinct structural and functional profiles under standardized film-forming conditions. CM-Crb and Ch-Crb, although both crab-derived and with similar MW values (~100 kDa), showed different ATR-FTIR profiles and significantly different EAB, supporting the contribution of molecular characteristics beyond chain length to film behavior.

Antibacterial inhibition was predominantly observed for the crab-derived CM-Crb and Ch-Crb films and increased with chitosan concentration, whereas CM-Shr, Ref-H, and Ref-L showed no detectable inhibition beyond the disk diameter under the tested conditions. CM-Crb combined a relatively high tensile strength (52.1 ± 4.3 MPa) with the highest EAB (3.7 ± 0.3%) and showed the highest measured UV blocking at 340 nm (35%), although the optical results were descriptive.

Overall, the distinct film properties were associated with the physicochemical profiles of the starting chitosans, although the individual contributions of biological source, extraction approach, MW, DDA, and other material characteristics cannot be independently resolved from the present experimental design. Thus, the results highlight the importance of considering the overall physicochemical characteristics of the starting chitosan, together with formulation conditions, when designing bioactive films. The hybrid chemical–microbiological approach provided chitosans capable of forming films with functional mechanical and antibacterial performance, supporting its potential as an alternative route for the valorization of crustacean residues. However, its economic and scale-up advantages over conventional chemical extraction remain to be evaluated. Future studies should complement the agar disk-diffusion assay with viable-count approaches to further characterize antibacterial performance and evaluate key barrier properties, particularly water vapor and oxygen permeability, as well as carbon dioxide permeability where relevant. Together with migration and safety assessments and performance evaluation in actual food systems, these additional characterizations will help define the potential of these films for food-packaging applications.

## Figures and Tables

**Table 1:** Origin, extraction approach, and physicochemical characteristics of experimentally produced chitosans.

Chitosan	Origin	Extraction	DDA (%)	MW (kDa)	Ash (%)	Total Nitrogen (%)	Moisture (%)
CM-Crb	Crab	Chemical– microbiological	75.1 ± 2.3	100	0.4 ± 0.1	6.9 ± 0.1	6.7 ± 0.2
CM-Shr	Shrimp	Chemical– microbiological	80.8 ± 0.3	110	0.2 ± 0.2	6.6 ± 0.1	6.6 ± 0.3
Ch-Crb	Crab	Chemical	80.2 ± 2.1	100	0.2 ± 0.1	6.5 ± 0.1	6.9 ± 0.4

Values for DDA, ash content, total nitrogen, and moisture content are expressed as mean ± SD. MW represents the viscosity-average molecular weight.

**Table 2:** Thermal degradation temperatures and associated mass losses of chitosan films.

Sample	Initial Moisture Loss		Intermediate Thermal Event		Main Polymer Degradation		High-Temperature Residual Degradation	
	Td (°C)	Δw (%)	Td (°C)	Δw (%)	Td (°C)	Δw (%)	Td (°C)	Δw (%)
CM-Crb	83.7	8.8	198.3	18.1	253.0	36.5	673.6	26.0
CM-Shr	86.4	8.5	187.5	13.4	264.1	38.6	597.7	29.9
Ch-Crb	78.2	10.7	194.4	15.3	252.8	39.2	580.0	15.2
Ref-H	82.9	8.9	190.4	14.5	272.6	39.9	570.4	31.9

**Table 3:** Mechanical test results of chitosan films prepared from film-forming solutions containing 1.5% (*w*/*v*) chitosan and plasticized with glycerol at 30% (*w*/*w*, based on chitosan dry weight).

Sample	Thickness (mm)	Tensile Strength (MPa)	Elongation at Break (%)	Young’s Modulus (GPa)
CM-Crb	0.07 ± 0.00	52.1 ± 4.3 ab	3.7 ± 0.3 a	3.4 ± 0.3 a
CM-Shr	0.07 ± 0.00	47.3 ± 4.6 ab	2.3 ± 0.4 b	2.9 ± 0.7 a
Ch-Crb	0.07 ± 0.01	37.6 ± 6.6 b	2.5 ± 0.4 b	2.2 ± 0.6 a
Ref-H	0.08 ± 0.02	56.4 ± 7.2 a	3.2 ± 0.2 ab	3.5 ± 0.4 a
Ref-L	0.07 ± 0.00	42.2 ± 6.1 ab	2.5 ± 0.2 b	2.2 ± 0.3 a

Values are expressed as mean ± standard deviation. Different letters within the same column indicate significant differences among chitosan films according to one-way ANOVA followed by Tukey’s HSD test (*p* < 0.05). For film thickness, a standard deviation of 0.00 mm indicates that no variation was observed among replicate measurements at the measurement precision used.

**Table 4:** Optical properties of chitosan films prepared from film-forming solutions containing 1.5% (*w*/*v*) chitosan and plasticized with glycerol at 30% (*w*/*w*, based on chitosan dry weight).

Sample	Thickness (mm)	Total Light Transmittance (%)	UV Blocking at 340 nm (%)	Diffused Light (%)
CM-Crb	0.07	88.4	35	4.4
CM-Shr	0.07	89.8	18	6.3
Ch-Crb	0.07	84.8	18	5.7
Ref-H	0.07	87.0	17	6.2

## Data Availability

The data supporting the findings of this study are available from the corresponding author upon reasonable request.
